# Association of COVID-19 Pandemic with Newly Diagnosed Anorexia Nervosa Among Children and Adolescents in Japan

**DOI:** 10.3390/medicina61030445

**Published:** 2025-03-03

**Authors:** Yoshifumi Fukuya, Keitaro Miyamura, Tomoyuki Funatogawa, Taiju Yamaguchi, Naoyuki Katagiri, Takahiro Nemoto

**Affiliations:** 1 Department of Neuropsychiatry, Faculty of Medicine, Toho University, Tokyo 143-8541, Japan; yoshifumi.fukuya@med.toho-u.ac.jp (Y.F.); tomoyuki.funatogawa@med.toho-u.ac.jp (T.F.); taiju.yamaguchi@med.toho-u.ac.jp (T.Y.); ktgrnoyk@med.toho-u.ac.jp (N.K.); 2 Center for Excellence in Thyroid Care, Kuma Hospital, Kobe 650-0011, Japan; miyamura@kuma-h.or.jp; 3 Department of Psychiatry and Implementation Science, Faculty of Medicine, Toho University, Tokyo 143-8541, Japan

**Keywords:** anorexia nervosa, COVID-19, eating disorders, childhood, adolescence

## Abstract

*Background and Objectives*: The COVID-19 pandemic globally has negative effects on mental health. Research from Western countries, such as the US, Canada, Australia, and Europe, shows that the COVID-19 pandemic is associated with an increased trend of anorexia nervosa (AN) among children and adolescents. However, the trend after the pandemic in Eastern countries, including Japan, is not well-understood, and it remains unclear whether the pandemic is associated with the trend in these countries. This study aimed to examine the association between the COVID-19 pandemic and the newly diagnosed AN among children and adolescents in Japan. *Materials and Methods*: Using the nationwide multi-hospital database in Japan, we analyzed the clinical outpatient data in the departments of pediatrics, psychosomatic medicine, and psychiatry. The participants comprised children and adolescents aged 7–19 years newly diagnosed with AN from January 2017 to January 2022. An interrupted time series analysis was used to compare the trends of newly diagnosed AN before and after the COVID-19 pandemic. Estimating the changes in the trends over the pandemic was performed using a Poisson regression model. *Results*: The total cases of newly diagnosed AN were 41 cases diagnosed in 38 months (1.08 cases per month) before the pandemic and 34 in 23 months (1.48 cases per month) after the pandemic. Notably, in the 7–14 age group, the cases per month increased from 0.74 to 1.13 cases per month over the period. Before the pandemic, a decrease in cases was observed (Incidence Rate Ratio [IRR] = 0.961; 95% CI = 0.932–0.990). Conversely, the after-pandemic period showed a contrasting trend, with increased cases (IRR = 1.096; 95% CI = 1.032–1.176). Furthermore, the number and cases per month in boys increased over the period, from 1 to 5 cases and from 0.03 to 0.22 cases per month, compared to girls from 28 to 26 cases and from 1.05 to 1.26 cases per month. *Conclusions:* Our findings suggest that sociocultural differences at a national level may not affect the trend of AN after the pandemic. The pandemic and lifestyle changes after the pandemic occurred in both Western and Eastern countries. Considering that, individual, parental, and peer factors related to the pandemic and the consequent lifestyle changes may be more associated with the development of AN. Further research in different countries is needed to elucidate the mechanisms of AN and the long-term impact of the pandemic.

## 1. Introduction

Anorexia nervosa (AN) is one of the mental health problems negatively affecting both individuals and societies [1]. The onset of AN often starts in the age of adolescence [2], and AN has negative impacts on physical and psychological development during adolescence [3,4]. Also, individuals with AN have a higher mortality risk and an increased risk of suicide [5]. Furthermore, research from the US has reported that AN is a non-negligible economic burden on society [1,6]. Given these features, addressing AN is required to minimize these adverse impacts. Previous research has demonstrated several preventive measures, such as nutritional, physical activity, psychological, psychosocial, and physical therapy interventions [7,8]. However, eating disorders, including AN, are multifactorial problems [9]. Accordingly, specific interventions to reduce risk factors or promote protective factors have limitations in intervening AN. Then, there is an urgent need to elucidate triggering factors related to the onset of AN for the preventative intervention.

Previous research has shown that the onset of AN is associated with not only individual and parental factors but also social factors [10,11]. According to prior literature, there are cultural differences in the psychopathology of AN between Western and Eastern countries [12,13]. Furthermore, each Asian country is found to have different characteristics in the development of AN [14]. Thus, sociocultural features in each country should be considered in order to understand the etiology of AN.

Since December 2019, COVID-19 has spread globally, and the pandemic negatively affects mental health for all ages. Research has shown that the pandemic increased the risk of mental health problems like depression, anxiety, and suicide [15,16,17]. Furthermore, previous studies have reported increased trends of AN among child and adolescent patients for one year after the pandemic, mainly in Western countries such as the US, Canada, Australia, and Europe [18,19,20,21]. Prior studies and reviews indicated that social isolation, school closure, an increase in the use of social media, and disruptions to routine changes as lifestyle changes after the pandemic may lead to eating disorders [19,22,23,24].

In Japan, on 15 January 2020, the first case of COVID-19 infection was reported; since then, the infection has rapidly spread across Japan. On 27 February, the government requested nationwide school closure, including elementary, junior high, and high school, to address the spread of the COVID-19 infection. Indeed, school closures began on 2 March 2020. On 7 April 2020, the Japanese government declared a state of emergency for seven prefectures, and on 16 April, the declaration was extended to cover all prefectures. The declaration enables prefectural governors to request residents in the areas to stay at home except for essential activities. Responding to the declaration, 91% and 80% of schools were closed at the end of April and May 2020, respectively [25]. After the school closure, the lifestyles among school-aged children and adolescents drastically changed: They stayed at home and received online lectures and lost opportunities to participate in school and extracurricular activities and to interact with peers face-to-face. At least, the lifestyle changes have affected until the sixth wave of the COVID-19 infection, January to March 2022 [26].

Given the global impacts of the pandemic, the pandemic may affect the trend of AN among children and adolescents in other countries, such as Eastern countries, including Japan. To date, it remains unclear whether the trend of AN among children and adolescents increases or not after the COVID-19 pandemic in Eastern countries. If the different trends between Western and Eastern countries exist despite the global pandemic, sociocultural differences between Western and Eastern countries may play a preventive role in AN. Thus, evidence from Eastern countries may contribute to elucidating the triggering factors of AN and the mechanisms of the development.

To date, the prevalence of AN in Eastern countries has been lower than in Western countries [27]. Prior reports demonstrated that the prevalence in China and Japan was 1.05% and 0.43%, respectively [28,29,30], compared to Western countries, which were 1–4% in European countries [31]. Given the differences in the prevalence, sociocultural differences between Eastern and Western countries have been pointed out to affect the differences, although these factors have not been well understood [27]. Furthermore, Japan has a universal health insurance system [32]. Also, the Japanese government and prefectures support medical expenses for children and adolescents. Accordingly, children and adolescents with initial symptoms of disordered eating behavior can easily access the department of pediatrics, psychosomatic medicine, or psychiatry and receive medical care. Early intervention to the symptoms plays a preventive role in developing AN [33,34]. Thus, considering these sociocultural features in Japan, we hypothesize that the trend of AN in Japan may be different from that in Western countries.

A previous study in Japan showed the association between the pandemic and an increased trend of eating disorders among children and adolescents [35]. However, it is still unknown whether the pandemic is associated with AN among children and adolescents in Japan. Then, this study aimed to investigate the association between the COVID-19 pandemic and the newly diagnosed AN among children and adolescents in Japan, using a multi-hospital database.

## 2. Materials and Methods

### 2.1. Study Design and Data Sources

This study was a quasi-experimental study using multi-hospital electronic medical records from the Real World Data (RWD) database. A quasi-experimental design would be useful when random allocation is not feasible due to ethical and practical problems [36]. The RWD database is maintained by the Health, Clinic, and Education Information Evaluation Institute (HCEI, Kyoto, Japan) and managed by Real World Data Co., Ltd. (Kyoto, Japan). The RWD database is a nationwide database containing the medical records of over 20 million patients from approximately 200 medical centers in 2021. The database includes information about age, gender, diagnosis, the diagnosis date, the geographical locations and the department of medical centers, and the number of inpatient beds in the centers where patients were diagnosed. Diagnoses were based on the ICD-10 (the Tenth Revision of the International Classification of Diseases) codes.

### 2.2. Participants

This study included patients aged 7–19 years who were diagnosed with anorexia nervosa (ICD-10 code: F50.0) at the medical centers in the RWD database from 1 January 2017 to 31 January 2022. We limited our study to the patients diagnosed in the outpatient departments of pediatrics, psychosomatic medicine, and psychiatry. As for the medical centers, we included the centers registered in the RWD database before January 2017. We excluded the centers in which electronic medical records were continuously unavailable until 31 January 2022.

### 2.3. Statistical Analysis

An interrupted time series analysis was used to evaluate the association between the COVID-19 pandemic and the newly diagnosed AN in children and adolescents. In this analysis, we compared the trends of newly diagnosed AN cases before and after the pandemic. We defined school closure, March 2020, as the onset of the pandemic because lifestyles changed significantly after the school closure [25,26]. This study used one month as the unit of time during the observation period. The time variable and the interaction term before and after the pandemic were added to estimate the trend changes. Our data comprised 38 months in the before-pandemic period and 23 months in the after-pandemic period. The trends were examined using a Poisson regression model with overdispersion adjustment though a scale parameter [37]. We found no seasonal patterns in the trends and no autocorrelation in the model. All analyses were performed using STATA version 15 (Stata Corp. 2017, College Station, TX, USA).

## 3. Results

Table 1 shows the demographic characteristics of newly diagnosed AN in children and adolescents aged 7–19 years. The total number of newly diagnosed AN before and after the COVID-19 pandemic is 41 cases (1.08 cases per month) and 34 cases (1.48 cases per month), respectively. As for gender, the number of female cases is higher than male in both before- and after-pandemic periods. The number of male cases per month increases from 0.003 (one case) in the before-pandemic period to 0.22 (five cases) in the after-pandemic period. The number of female cases per month is 1.26 (29 cases) in the after-pandemic period, compared to 1.05 (40 cases) in the before-pandemic period. Regarding the age groups, the number of newly diagnosed cases in the 7–14 age group is higher than in the 15–19 age group in both periods. The number of cases aged 7–14 years in the before-pandemic period is 28 cases, almost equal to 26 cases in the after-pandemic period. The number of cases per month in this age group after the pandemic increased approximately 1.5 times compared to before the pandemic (1.13 from 0.74 cases per month). For children and adolescents aged 15–19 years, the number of cases and cases per month is almost the same in both periods. As for the departments, in both periods, most new cases of AN were diagnosed in the department of psychosomatic medicine, followed by pediatrics and psychiatry. In the department of pediatrics, the number of cases per month after the pandemic increased to 0.48 cases per month (11 cases), the most, approximately 1.8 times, compared to 0.26 cases per month (10 cases) before the pandemic. As for the number of inpatient beds in the medical centers, the number of new cases in the centers with 100–299 beds is the most among all centers in both periods. The number of cases per month in medical centers with 100–299 and ≥500 beds after the pandemic increased almost 1.5 times and 1.3 times, respectively, compared to before the pandemic (1.04 from 0.76 cases per month and 0.39 from 0.029 cases per month, respectively). In the regions of the medical centers, the number of new cases in the Chuubu region is highest in both the before- and after-pandemic periods. In the Tohoku region, the number of new cases is ten cases before the pandemic period and nine cases after the pandemic period (0.26 and 0.39 cases per month, respectively). The number of new cases in the Kinki and Kanto regions is the same in both periods.

**Figure 1:**
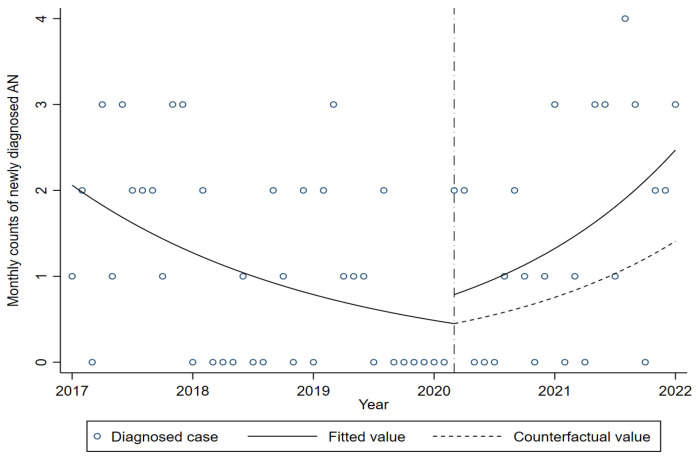
Trends in newly diagnosed cases of AN aged 7–19 years. The short-dashed line indicates the period of school closure, March 2020. The dashed line shows the counterfactual trend after the COVID-19 pandemic.

Figure 1 shows the monthly trends of newly diagnosed AN cases aged 7–19 years. The figure illustrates the decreasing trend in the before-pandemic period. After the pandemic, the figure shows that the trend in newly diagnosed cases increased faster than the counterfactual trend.

Table 2 shows the changes in the slope and level of newly diagnosed AN cases aged 7–19 years. In the before-pandemic period, a significant decrease in the new cases was observed (incidence rate ratio [IRR] 0.961; 95% confidence interval [CI] 0.932–0.990). After the pandemic, there was no significant change in the level of the trend (IRR 1.753; 95% CI 0.578–5.319). In the after-pandemic period, a significantly increased trend in the new cases was observed (IRR 1.096; 95% CI 1.032–1.176).

## 4. Discussion

This study examined the association between the COVID-19 pandemic and the newly diagnosed AN among children and adolescents using the multi-hospital database in Japan. This study showed that the pandemic was associated with an increased trend in newly diagnosed AN cases after the pandemic. Our findings suggest that the pandemic may affect the development of AN among children and adolescents in Japan.

Our findings are consistent with prior studies in Western countries, such as the US, Canada, Australia, and Europe [18,19,20,21]. Our results also show that the number of AN cases per month, notably in the younger age group (7–14 years old), increased after the pandemic. The findings are in line with a previous study in Canada, which showed an increased number of hospitalized AN aged under 15 years old after the first wave of the pandemic [18]. Consequently, our findings suggest that the impact of the pandemic on AN among children and adolescents may be no different between Eastern and Western countries. Given the findings, sociocultural differences at a national level may not be associated with an increased trend of AN after the pandemic. In Japan, after the pandemic, lifestyles among school-aged children and adolescents drastically changed due to school closure. Thus, the pandemic and the consequent lifestyle changes were common phenomena in both Western and Eastern countries. Considering that, individual, parental, and peer factors through the pandemic and the consequent lifestyle changes may be more associated with the development of AN.

This study demonstrated more increases in the number of AN cases and its cases per month among boys after the pandemic, compared to girls. Previous studies demonstrating the impact of COVID-19 in Western countries did not report an increase in the trend of cases among boys. However, several epidemiological studies before the pandemic showed the recent increased frequency of eating disorders among boys in Australia, Brazil, and Switzerland [38,39,40,41]. Thus, an increased trend of boys in our findings may be related to not only the COVID-19 pandemic but also the recent trend in such Western countries. Further empirical research is needed to elucidate the trend of boys’ changes over the period and whether that in Japan has similarities to Western countries.

The present findings are also in accordance with a previous study on the trend of eating disorders among children and adolescents in Japan [34]. The previous study showed an increased trend of eating disorders coded by F50.0–F50.9. This study also included the patients diagnosed in the adult departments and the departments not related to mental health, which may lead to an overestimation of the diagnoses [34]. In our study, we focused on the patients diagnosed with AN (F50.0) in the departments of pediatrics, psychosomatic medicine, and psychiatry to improve the reliability of the AN diagnosis among children and adolescents. Our findings may contribute to showing the current trend of AN among children and adolescents in Japan, which in turn provides new evidence on the association of the COVID-19 pandemic with AN among children and adolescents from Eastern countries.

Regarding the mechanisms of the association between the pandemic and AN among children and adolescents, there may be several explanations based on individual, parental, and peer factors. First, as for individual factors, anxiety related to the pandemic may affect the onset of AN. Recent research has shown the increased prevalence of anxiety symptoms in children and adolescents during the pandemic [42]. According to prior studies, anxiety increases the risk of AN [43,44]. Furthermore, research has shown that individuals with perfectionism have a higher level of anxiety when they perceive psychological distress [45]. Indeed, perfectionism is a well-known risk factor for AN [46,47]. Moreover, recent research reports that psychological distress, including anxiety, related to COVID-19 mediates the relationship between perfectionism and disordered eating behavior [48]. Thus, an increased anxiety after the pandemic may trigger the development of AN; particularly, children and adolescents with perfectionism may be more likely to be at higher risk of AN after the pandemic.

Furthermore, previous research has demonstrated several features, such as intolerance of uncertainty, heightened sensitivity to uncontrollability, psychological inflexibility, and reduced social-emotional skills, are associated with eating disorders [39,49,50,51,52]. Moreover, prior studies demonstrated that these features are related to anxiety. For example, one study among undergraduate students indicated that anxiety related to COVID-19 was strongly associated with the pathology of eating disorders for individuals with a lower intolerance of uncertainty [53]. Furthermore, “uncontrollability worry” is the central manifestation in generalized anxiety symptoms among adolescents [54]. Indeed, the pandemic is an uncontrollable condition [55]. Thus, individuals with heightened sensitivity to uncontrollability may be more likely to have an increased risk of anxiety during the pandemic. Another study showed a positive relationship between psychological inflexibility and anxiety among children [56]. Furthermore, prior research showed that reduced social and emotional skills are associated with anxiety symptoms among children and adolescents [57,58]. Given the evidence, these features along with anxiety after the pandemic may lead to an increased risk of AN. Thus, children and adolescents with these features may be more vulnerable to the pandemic.

Second, relationships with parents may have a prominent effect on mental health among children and adolescents after the pandemic. Family memberships have had more time to spend together at home due to teleworking and school closure during the pandemic. According to previous studies, authoritarian and neglectful parenting styles are associated with disordered eating behavior [59,60]. Furthermore, recent research demonstrates that parents’ distress during the lockdown is associated with poorer parenting and the decreased quality of the parent–child relationships [61]. Given the lifestyle changes after the pandemic, particularly children and adolescents with authoritarian and/or neglectful parents may be more likely to increase the likelihood of developing AN.

Third, social relationships with peers due to lifestyle changes may affect the development of AN during the COVID-19 pandemic. According to research, adolescents were more concerned about peer relationships, loneliness, and social isolation during the pandemic [62,63]. Recent research has reported a lower level of perceived peer social support among adolescents after the pandemic [64]. Furthermore, previous research has shown that peer social support helps young people to have positive images of one’s own bodies [65,66]. Other studies have reported that lower peer social support is linked to body dissatisfaction in adolescents [67]. Furthermore, recent studies indicate a higher rate of loneliness in adolescents after the pandemic [68,69]. Loneliness is known to be a risk factor for disordered eating behavior [70]. Thus, fewer social relationships with peers caused by lifestyle changes after the pandemic, for example, less perceived peer social support and higher loneliness, may lead to an increase in the risk of developing AN among children and adolescents during the pandemic.

In addition, a study in the UK reports that closer relationships with their parents are associated with lower levels of loneliness in adolescents during the lockdown [71]. Thus, individual, parental, and peer factors may mutually interact after the pandemic, which in turn may lead to the development of AN in children and adolescents.

Considering these potential explanations, the pandemic may be more likely to affect children and adolescents already having had the risk factors for AN, such as perfectionism and poor relationships with their parents and peers, which may lead to the increased trend of newly diagnosed AN after the pandemic; however, to date, the etiology and the mechanisms have not fully understood. Furthermore, it remains unclear how long the impact of the pandemic on AN will last. Thus, further studies are needed to elucidate the long-term impact of the pandemic in different regions and the mechanisms to prevent the development of AN in future crises.

### 4.1. Limitations

There are several limitations in this study. First, the RWD database we used is not national representative data. Furthermore, the differences in the department, the regions, and the number of inpatient beds are affected by the hospital registered to the database. These can be the selection bias. In response, we used multi-hospital data in this analysis and applied the data of the hospitals continuously registered in the database from January 2017 to January 2022 to minimize the bias. Second, regional differences in the COVID-19 prevalence may have different effects on the onset of AN in each region of Japan. However, the COVID-19 pandemic affected all prefectures in Japan. As a result, all public schools were closed in the first wave. After the school closure, lifestyles among children and adolescents changed in all prefectures, which suggests that the regional differences have little effect on the development of AN in Japan. Third, differences in the educational policy of each prefecture may differently affect the development of AN among children and adolescents. Although the board of education in local areas decided to close schools and school curricula in accordance with the epidemic in the regions, the lifestyle changes continued during the observed period. Fourth, the database does not include information about patients already diagnosed as AN in other medical centers that have not been registered in the RWD database, which may lead to overestimations of the effects of the pandemic. Fifth, the database also does not include information about the severity of AN patients, such as BMI, complications, and clinical impairment. Thus, we could not assess the trend of AN cases by the severity. Sixth, children and adolescent patients and their families may hesitate to visit hospitals because they avoid infection in public places. Even if the symptoms of AN occur before the pandemic, the patients may visit the medical center later after the pandemic. In that situation, the patients were counted as the cases diagnosed after the pandemic, which may cause an overestimated evaluation of the effects of the pandemic.

### 4.2. Implications

To the present, many studies have revealed the association of the COVID-19 pandemic with mental health problems among children and adolescents. Our findings indicate that the COVID-19 pandemic may globally affect the development of AN beyond sociocultural differences. However, it remains unclear whether the pandemic is associated with AN in other areas, such as Africa or developed countries. If there are no associations, sociocultural characteristics in these countries may mitigate the impact of the pandemic. Empirical research in such countries may elucidate sociocultural risk factors of AN among children and adolescents. This evidence contributes to developing personalized interventions based on sociocultural characteristics to prevent the development of AN.

Moreover, future endemics, epidemics, pandemics, and natural disasters have the potential to negatively change lifestyles. These lifestyle changes may intensify risk factors for AN at the individual, parental, and peer levels, thereby increasing the likelihood of developing AN. Children and adolescents who already have these risk factors may be more vulnerable to lifestyle changes, putting them at a higher risk for AN. When the symptoms of their disordered eating behavior occur due to lifestyle changes, it could be crucial for clinicians and health care providers to promptly assess and address the underlying risk factors [72]. Additionally, precision interventions that focus on each specific risk may help to minimize the impact of these lifestyle changes and prevent the onset of AN.

## 5. Conclusions

Our study found an association between the COVID-19 pandemic and AN among children and adolescents in Japan as well as Western countries. Individual, parental, and peer factors related to the pandemic and the consequent lifestyle changes may be more associated with the development of AN. The pandemic may be more likely to affect those who potentially have these risk factors, which may lead to their disordered eating behavior. In addition, early adolescents and boys may have more risk of developing AN. Further research is required to prevent AN among children and adolescents for future pandemics.

## Figures and Tables

**Table 1:** Demographic characteristics of newly diagnosed AN cases aged 7–19 years.

	Before-Pandemic Period Jan. 2017–Feb. 2020 (38 Months)		After-Pandemic Period Mar. 2020–Jan. 2022 (23 Months)	
	No. of Cases	No. of Cases Per Month	No. of Cases	No. of Cases Per Month
Total No.	41	1.08	34	1.48
Gender				
Male	1	0.03	5	0.22
Female	40	1.05	29	1.26
Age groups				
7–14	28	0.74	26	1.13
15–19	13	0.34	8	0.35
Department				
Pediatrics	10	0.26	11	0.48
Psychosomatic Medicine	30	0.79	22	0.96
Psychiatry	1	0.03	1	0.04
No. of inpatient beds in medical centers				
100~299	29	0.76	24	1.04
300~499	1	0.03	1	0.04
500≤	11	0.29	9	0.39
Regions of medical centers				
Chuubu	29	0.76	23	1.00
Tohoku	10	0.26	9	0.39
Kinki	1	0.03	1	0.04
Kanto	1	0.03	1	0.04

**Table 2:** Trend changes in newly diagnosed cases of AN before and after the COVID-19 pandemic.

	Newly Diagnosed Case of AN Aged 7–19 Years		
	Incidence Rate Ratio	95% Confidence Interval	*p*-Value
Slope before the pandemic	0.961	0.932 to 0.990	0.009
Level changes after the pandemic	1.753	0.578 to 5.319	0.322
Slope after the pandemic	1.096	1.032 to 1.176	0.003

## Data Availability

The data that support the findings of this study are available from the Real World Data Co., Ltd., but restrictions apply to the availability of these data, which were used under license for the current study, and so are not publicly available.
